# Wheat Gluten

**Published:** 1877-05

**Authors:** 


### *WHEAT GLUTEN.*

In Paris we visited Mr. Conor's gluten factory. He uses common white flour, and as white flour is not very rich in gluten, he has to charge three francs a pound for the product. Its price in New York, duty paid and profit added, is $1 per pound. It is a valuable food, because rich in nutritive matter and quite free from starch. The process of the Health Food Company here, is to moisten and rub off the hull of the grain, reduce the soluble remainder by the cold blast, and then precipitate the gluten from the starch, in water. By using machinery and steam power they are able to extract the gluten for a little less than eleven cents per pound, with wheat at its present high figure. The rapidity with which worn-out dyspeptics, constipated sufferers, and diabetics are made well by the use of this noblest of foods, is well-nigh marvelous. We have recommended it to some of the worst sufferers from dyspepsia whom we have seen of late—old-fashioned Grahamites—the very hardest patients in the world to bring around in chronic forms of disease, because uniformly so weakened in vital power through long-continued depletion, as to leave nothing to build on—and have been greatly gratified by the result. Digestion is restored to its normal state by feeding directly the ganglionic nerve centers of the intestines. We cordially commend this as the best food within our knowledge. Its sale has been greatly increased within the past winter. When the Health Food Company began its manufacture, two years ago, they had only one small tank, capable of precipitating forty pounds a day. They now employ ten large tanks, with an aggregate capacity of a thousand pounds daily.

And here we will repeat a remark just made to us by the gentleman who invented the "Fire on the Hearth" stove, which has kept us so delightfully comfortable during the cold term—Mr. Phillips, of 107 Fulton Street. He declares this gluten to be the food of foods for the sedentary man, woman, or child. In his opinion dyspepsia would not be known if gluten were used daily by all.

